# International Intercomparisons of Photometric Base Units

**DOI:** 10.6028/jres.092.033

**Published:** 1987-10-01

**Authors:** Klaus D. Mielenz

**Affiliations:** National Bureau of Standards, Gaithersburg, MD 20899

**Keywords:** candela, international, luminous flux, luminous intensity, photometry

## Abstract

In order to evaluate the worldwide consistency of practical implementations of the 1979 redefinition of the candela, the Consultative Committee for Photometry and Radiometry (CCPR) has conducted an international intercomparison of photometric base units. The intercomparison showed 0.8% agreement (one standard deviation) of independent luminous-intensity scale realizations by 15 national laboratories, and 0.6% agreement of luminous-flux scale realizations by 11 laboratories. The NBS candela and lumen agreed with the world mean within quoted uncertainty limits, and were shown to be consistent with one another within 0.5%.

In 1979 the 16th General Conference on Weights and Measures (CGPM) accepted a recommendation by the Consultative Committee for Photometry and Radiometry (CCPR) and redefined the SI unit of luminous intensity as follows:

“The candela is the luminous intensity, in a given direction, of a source that emits monochromatic radiation of frequency 540×10^12^ hertz and has a radiant intensity in that direction of 1/683 watts per steradian.”

“The candela so defined is the base unit applicable to photopic quantities, scotopic quantities, and quantities to be defined in the mesopic domain.”

This definition abrogated the platinum-point blackbody as the universal “primary standard of light” which defined the candela between 1948 and 1979. It established for the first time an exact numerical relationship between photometric and radiometric quantities. According to the current system of physical photometry that has been adopted by the International Commission on Illumination (CIE), this relationship is

$$Q_{v}=K_{m}\;\int \;d\lambda \;(dQ/d\lambda )\;V\;(\lambda ),$$

where *Q_v_* and *Q* are generalized luminous and radiant quantities, *V*(λ) is the relative spectral luminous efficiency of the 1931 CIE standard observer, and *K*_m_ is the maximum spectral luminous efficacy which has been fixed as 683 lm/W by the new definition of the candela. The above expression holds for photopic light adaptation. Equivalent expressions, with *K*_m_ and *V*(λ) replaced by appropriate other quantities as defined by the CIE, apply for scotopic and mesopic adaptation.

The main practical consequence of the 1979 candela definition and the CIE equations is that they allow choices of physical methods for realizing photometric scales. For example, lamps calibrated as spectroradiometric standards can also be used as photometric standards once the above integral has been evaluated numerically. An alternative approach is the realization of photometric scales based on absolute detectors fitted with optical filters, so that the absolute spectral responsivity of the detector/filter combination represents the spectral luminous efficiency function *K*_m_ *V*(λ).

The first of these approaches has been chosen by the National Bureau of Standards. Since 1979, NBS photometric standards have been derived from the NBS scale of spectral irradiance. The starting point of this derivation is the use of a gold-point blackbody to realize the 1968 International Practical Temperature Scale (IPTS-68), which is used in turn to measure the temperature of a variable temperature blackbody. The latter provides a scale of spectral radiance, which is then converted to a scale of spectral irradiance. All of the steps necessary to implement this measurement chain are performed on the NBS Facility for Automatic Spectroradiometric Calibrations (FASCAL). The NBS scale of spectral irradiance is maintained in a group of four 1000 W quartz halogen lamps. The primary calibration of the photometric working standards maintained at the Bureau is performed by making spectral irradiance measurements on FASCAL against these four quartz halogen lamps. The above integral is then evaluated using the adopted values of *K*_m_ and *V*(λ) recommended by the CIE.

The detector approach to realizing the new candela has been adopted by several other national standardizing laboratories. Electrical substitution radiometers as well as absolute standard silicon photodiodes have been used in these scale realizations. Experimental photometric measurements based on absolute silicon photodiodes have been made at NBS in collaboration with the Hungarian Academy of Sciences and Office of Measures. These measurements have shown an over-all agreement at the 0.5% level with the above mentioned NBS photometric scale derived from blackbody based spectral irradiance measurements.

Following the redefinition of the candela, the CCPR allowed a period of three years for the realization of the new unit in different countries. In 1982, it organized an international intercomparison of luminous intensity and luminous flux measurements for two purposes. The first, scientific, purpose was to evaluate the world-wide consistency of diverse approaches to photometric scale realizations based on the new definition. The second purpose was to establish, for commercial reasons, the relationships between the photometric units maintained and disseminated by different national laboratories. The intercomparison, which took place during the 1984–86 period, was carried out under the following guidelines:

The participating national laboratories made absolute measurements based on independent scientific realizations of the new candela definition.

The International Bureau of Weights and Measures (BIPM) at Sevres, France, served as the convening laboratory. Since the BIPM did not have the facilities for independent scale realizations, it compared the scientific values reported by the participating laboratories to the values assigned to primary BIPM standard lamps based on means of previous intercomparisons.

Two types of gas-filled incandescent lamps (Osram Wi41G and NPL/GEC lamps) were selected for the luminous intensity measurements. Each national laboratory calibrated six lamps of either type, or four of each. The lamps chosen for the luminous flux intercomparison were gas-filled, high color temperature GEC lamps. Each laboratory measured six of these lamps. All lamps were operated at color temperatures of 2800 K±30 K, under constant current conditions, and with fixed polarity.

After a first round of measurements, the participating laboratories shipped their lamps to BIPM for comparison with the photometric standards maintained there. The BIPM then returned the lamps to the participants for repeat measurements.

The results of the intercomparison were presented at the 11th Session of the CCPR in October 1986. They are summarized in table 1.

The spread in the candela and lumen values reported by the different national laboratories was slightly better than in previous intercomparisons. Because of the wider range of techniques now used for realizing photometric scales, this was seen as giving confidence to the new definition of the photometric base unit. Accordingly, the CCPR recommended

“that, by 1st July 1987, national laboratories make any necessary adjustment to the values attributed to their standards used for representing and disseminating the candela and the lumen in order to make them consistent with the definitions of these units,”

“that, by the same date, these laboratories advise the BIPM of the magnitudes of the adjustments made and of their best estimates of the differences between their adjusted values and the mean values of the 1985 comparison.”

Since NBS photometric scales have been based on the new candela definition since 1979, an adjustment of these scales was not necessary.

The intercomparison revealed a discrepancy in the photometric scales maintained at the BIPM. According to the average quotients shown on line 2 of table 1, the values attributed to the BIPM standards of luminous intensity were 1% lower than those corresponding to the mean candela of the intercomparison, and the values attributed to the BIPM standards of luminous flux were 0.7% greater than those corresponding to the mean lumen of the intercomparison. Since the BIPM units dated back to separate intercomparisons of luminous intensity and luminous flux measurements that were conducted in 1961 and 1952, the CCPR considered it mandatory to remove the 1.7% difference in the BIPM candela and lumen, and thus recommended

“that the values attributed to the standards maintained by the BIPM as representing the candela and the lumen be adjusted, with effect from 1st January 1987, so as to conserve and disseminate the mean result of the 1985 comparison of realizations of the candela and the lumen.”

The BIPM has since announced that this adjustment has been made.

With regard to the photometric scales maintained at NBS, it may be seen from table 1 that the ratios of the NBS luminous intensity and luminous flux values to the world mean of the intercomparison were 0.990/0.985=1.005 and 1.007/0.997=1.010. These differences fall well within the quoted uncertainties of 1.0% and 1.4%, respectively, for routine NBS luminous intensity and luminous flux calibrations. Nonetheless, research will be undertaken to investigate a possible deviation of the NBS lumen from the world mean. It should be noted, however, that the intercomparison showed the NBS candela and lumen to be consistent with each other to 0.5%.

## Figures and Tables

**Table 1:** Results of the international intercomparison for the candela and lumen values.

	Candela	Lumen
Number of participating national laboratories:	15	11
Average quotient of BIPM value (cd or lm) to values obtained by participating laboratories:	0.990	1.007
Standard deviation of quotients:	0.77%	0.58%
Quotient of BIPM and NBS values:	0.985	0.997

